# Two New Lanostane Triterpenoids from the Branches and Leaves of *Polyalthia oblique*

**DOI:** 10.3390/molecules19067621

**Published:** 2014-06-06

**Authors:** Liu-Kai Wang, Cai-Juan Zheng, Xiao-Bao Li, Guang-Ying Chen, Chang-Ri Han, Wen-Hao Chen, Xiao-Ping Song

**Affiliations:** Key Laboratory of Tropical Medicinal Plant Chemistry of Ministry of Education, College of Chemistry and Chemical Engineering, Hainan Normal University, Haikou 571158, Hainan, China

**Keywords:** *Polyalthia obliqua*, triterpenoid, antibacterial activity

## Abstract

Two new lanostane triterpenoids, 20-hydroxyeuphorbol-7-one (**1**) and 15α-hydroxyeuphorbol-7,11-dione (**2**), together with four known triterpenoids, euphorbol-7-one (**3**), friedelin (**4**), stigmast-4-ene-6*α*-ol-3-one (**5**), stigmasta-4-en-3,6-dione (**6**), were isolated from ethanol extract of the branches and leaves of *Polyalthia obliqua*. The structures of **1** and **2** were elucidated on the basis of extensive spectroscopic analysis and comparisons with related known compounds. Antibacterial activities of two new compounds and four known compounds were tested.

## 1. Introduction

**Figure 1:**
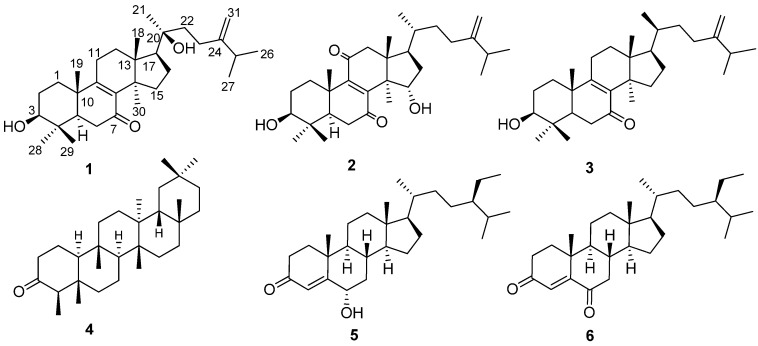
Structures of compounds **1**–**6**.

There are about 120 species of *Polyathia* in the world, of which only 17 species are distributed in China and *Polyalthia obliqua* is one of the seven *Polyalthia* species growing on Hainan Island, P.R. China. The genus is known for its folk medicine applications against a number of ailments such as stomach ache, dysmenorrhea and pharynx neurosis [1]. There were a series of interesting compounds isolated from the genus *Polyathia,* such as daphnane diterpenoids, alkaloids and phenanthrenes [2,3]. The lanostane triterpenoid suberosol isolated from *P. Suberosa* showed good anti-HIV activity [4]. In our previous work on this genus, 10 compounds were isolated from the roots of *P. obliqua* like suberosol, marcanine A and so on [5]. In continuation of our research, the petroleum ether fraction of the EtOH extract of the branches and leaves of *P. oblique* showed antibacterial activity against the *Escherichia coli*, with the MIC value of 20 μg/mL. In order to study on bioactive components from the branches and leaves of *P. oblique*, two new lanostane triterpenoids, namely, 20-hydroxyeuphorbol-7-one (**1**); and 15α-hydroxyeuphorbol-7,11-dione (**2**); together with four known triterpenoids, euphorbol-7-one (**3**); friedelin (**4**); stigmast-4-ene-6*α*-ol-3-one (**5**); stigmasta-4-en-3,6-dione (**6**) (Figure 1) were isolated from the EtOH extract of the branches and leaves of *P. oblique*, collected from Hainan province. This paper described the isolation, structural characterization and biological activities of these compounds.

## 2. Results and Discussion

The air-dried branches and leaves of *P. oblique* were extracted three times with 80% EtOH at room temperature. Repeated chromatography on a petroleum ether fraction from EtOH extract afforded two new compounds **1**–**2** and four known compounds **3**–**6**.

Compound **1** was obtained as a white amorphous powder, and its HRESIMS in the positive mode revealed a peak at *m*/*z*: 471.3826 [M+H]^+^ indicative of the molecular formula of C_31_H_5__0_O_3_, indicating seven degrees of unsaturation. Its IR spectrum exhibited the presence of hydroxyl (3,439 cm^−1^) and carbonyl groups (1,642 cm^−1^).

**Figure 2:**
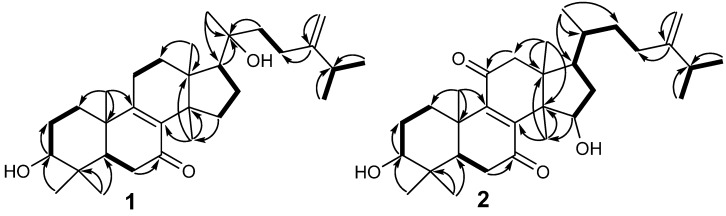
^1^H-^1^H COSY (▬) and Key HMBC (**→**) correlations of **1** and **2**.

In the ^1^H-NMR spectrum, six methyl singlet signals at *δ*_H_ 0.83 (3H, s), 0.88 (3H, s), 0.94 (3H, s), 1.00 (3H, s), 1.16 (3H, s) and 1.28 (3H, s), two methyl doublet signals at *δ*_H_ 1.01 (3H, d, *J* = 6.6 Hz) and 1.03 (3H, d, *J* = 6.6 Hz), one oxymethine signal at *δ*_H_ 3.26 (1H, dd, *J* = 11.6, 3.2 Hz) and two olefinic proton signals at *δ*_H_ 4.67 (1H, br s) and 4.73 (1H, br s), and a series of overlapped signals were observed. The ^13^C-NMR and DEPT spectra of **1** displayed 31 carbon signals which were assigned to eight methyls, ten methylenes, four methines, and nine quaternary carbons (including one carbonyl group at *δ*_C_ 199.2). The two olefinic carbon signals at *δ*_C_ 106.5 (CH_2_) and 156.3 (C) were characteristic of the exomethylene moiety. The other two olefinic carbon signals appearing at *δ*_C_ 138.5 (C) and 164.8 (C) corresponded to one double bond. The ^13^C-NMR chemical shifts at *δ*_C_ 75.3 (C) and 78.1 (CH) confirmed the presence of two oxygenated carbons. The aforementioned data indicated that **1** was most likely a lanostane triterpenoid with a C9 side-chain moiety at C-17 containing a CH_2_ substituent at C-24 [6]. Its ^1^H- and ^13^C-NMR spectra were very similar to those of the reported compound euphorbol-7-one (euphorbol-7-one is a lanostane triterpenoid, which has been isolated from the *Euphorbia sapinii* [7]), except for the disappearance of a methine signal at *δ*_H_ 1.43 (1H, m) in the ^1^H-NMR spectrum for **1**. Furthermore, in the ^13^C-NMR spectrum, the C-20 signal moved downfield significantly [*δ*_C_ 75.3 (C) in **1** *vs.* 36.4 (CH) in euphorbol-7-one], indicating a hydroxyl group was located at C-20. The location of the hydroxyl group at C-20 was further confirmed by the HMBC correlations from H-17, C-21, C-22 to C-20. The planar structure of **1** was further determined by the 2D NMR spectra including ^1^H-^1^H COSY and HMBC experiments (Figure 2).

**Figure 3:**
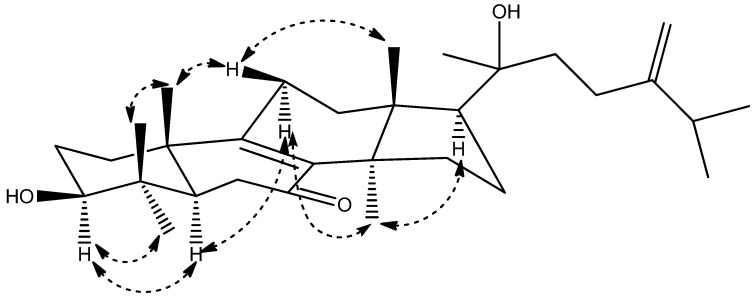
Key NOESY correlations of **1**.

The relative configuration of **1** was determined by extensive analysis of the coupling constants of ^1^H-NMR (Table 1) and NOESY correlations (Figure 3). The large coupling constant (*J* = 11.6 Hz) between H-3 and H*_β_*-2 clearly indicated that the 3-OH group was in equatorial *β*-position. The NOESY correlations (Figure 2) of H-3 with H-5 and H_3_-28, H-5 with H*_α_*-11, H_3_-30 with H*_α_*-11 and H-17, showed that H-3, H-5, H*_α_*-11, H-17, H_3_-28 and H_3_-30 were all in *α*-orientation, whereas H_3_-29 with H_3_-19, H_3_-19 with H*_β_*-11, H_3_-18 with H*_β_*-11 suggested that H*_β_*-11, H_3_-18, H_3_-19 and H_3_-29 were in *β*-stereochemistry. Because of the flexibility of the C-17 side chain, the relative configuration of C-20 was not determined. Thus compound **1** was determined to be 20-hydroxyeuphorbol-7-one, and the relative configuration of **1** is shown in Figure 2.

**Table 1:** NMR data for compounds **1**–**2** (*δ* in ppm J in Hz, in CDCl_3_, 400 and 100 MHz).

No.	1		2	
	*δ*_C_	*δ*_H_	*δ*_C_	*δ*_H_
1	34.9, CH_2_	1.46 (1H, m, H-*α*)	34.3, CH_2_	1.26 (1H, m, H-*α*)
		1.81 (1H, m, H-*β*)		2.84 (1H, m, H-*β*)
2	27.6, CH_2_	1.65 (1H, m, H-*α*)	27.7, CH_2_	1.74 (2H, m)
		1.75 (1H, m, H-*β*)		
3	78.1, CH	3.26 (1H, dd, 11.6, 3.2)	77.6, CH	3.28 (1H, dd, 11.2, 4.4)
4	39.1, C		38.9, C	
5	49.9, CH	1.61 (1H, overlapped)	49.9, CH	1.55 (1H, overlapped)
6	36.8, CH_2_	2.39 (1H, m, H-*α*)	35.6, CH_2_	2.55 (2H, m)
		2.44 (1H, m, H-*β*)		
7	199.2, C		205.6, C	
8	138.5, C		150.1, C	
9	164.8, C		154.8, C	
10	40.0, C		40.1, C	
11	22.9, CH_2_	1.80 (1H, m, H-*α*)	201.9, C	
		2.29 (1H, m, H-*β*)		
12	30.7, CH_2_	1.79 (1H, m, H-*α*)	52.2, CH_2_	2.82 (1H, d, 15.6, H-*α*)
		1.86 (1H, m, H-*β*)		2.86 (1H, d, 15.6, H-*β*)
13	45.5, C		48.2, C	
14	48.1, C		48.0, C	
15	31.6, CH_2_	1.77 (1H, m, H-*α*)	72.3, CH	4.34 (1H, dd, 14.4, 7.2)
		2.12 (1H, m, H-*β*)		
16	23.8, CH_2_	1.26 (1H, m, H-*α*)	33.7, CH_2_	1.55 (1H, m, H-*α*)
		2.25 (1H, m, H-*β*)		2.21 (1H, m, H-*β*)
17	51.0, CH	1.80 (1H, m)	48.4, CH	1.82 (1H, m)
18	17.4, CH_3_	0.83 (3H, s)	17.3, CH_3_	0.86 (3H, s)
19	18.6, CH_3_	1.16 (3H, s)	17.5, CH_3_	1.29 (3H, s)
20	75.3, C		36.3, CH	1.93 (1H, m)
21	26.3, CH_3_	1.28 (3H, s)	18.4, CH_3_	0.89 (3H, s)
22	42.8, CH_2_	1.56 (2H, m)	34.5, CH_2_	1.17 (1H, m, H-*α*)
				2.56 (1H, m, H-*β*)
23	29.1, CH_2_	1.26 (1H, m, H-*α*)	31.0, CH_2_	1.86 (1H, m, H-*α*)
		1.33 (1H, m, H-*β*)		2.07 (1H, m, H-*β*)
24	156.3, C		156.3, C	
25	34.0, CH	2.25 (1H, m)	33.9, CH	2.24 (1H, m)
26	22.1, CH_3_	1.01 (3H, d, 6.6)	22.0, CH_3_	1.01 (3H, d, 7.2)
27	22.0, CH_3_	1.03 (3H, d, 6.6)	21.9, CH_3_	1.03 (3H, d, 7.2)
28	27.6, CH_3_	1.00 (3H, s)	27.7, CH_3_	1.03 (3H, s)
29	15.4, CH_3_	0.88 (3H, s)	15.4, CH_3_	0.89 (3H, s)
30	25.4, CH_3_	0.94 (3H, s)	20.4, CH_3_	1.15 (3H, s)
31	106.5, CH_2_	4.67 (1H, br s)	106.6, CH_2_	4.65 (1H, br s)
		4.73 (1H, br s)		4.72 (1H, br s)

Compound **2** was obtained as a white amorphous powder. It has the molecular formula C_31_H_48_O_4_ group. from HRESIMS (*m*/*z*: 485.3627 [M+H]^+^, calcd 485.3625), indicating eight degrees of unsaturation. Its IR spectrum showed an absorption at 3441 cm^−1^ for hydroxyl groups and 1669 cm^−1^ for carbonyl. The general features of its NMR spectroscopic data (Table 1) were markedly similar to those of (3*β*)-3-hydroxy-24-methylenelanost-8-ene-7,11-dione (a lanostane triterpenoid isolated from the *Euphorbia humifusa* [8]). Detailed comparison of NMR data of these two compounds suggested that they had the same lanostane triterpenoid skeleton. The only significant difference in the ^1^H-NMR spectrum was the presence of a methine signal at *δ*_H_ 4.34 (1H, dd, *J* = 14.4, 7.2 Hz) in **2** instead of a methylene signal at *δ*_H_ 2.13–2.16 (2H, m) in (3*β*)-3-hydroxy-24-methylenelanost-8-ene-7,11-dione, and in the ^13^C-NMR spectrum a methine group at *δ*_C_ 72.3 (CH) for C-15 was observed in **2**, instead of a methylene group at *δ*_C_ 32.2 for C-15 in (3*β*)-3-hydroxy-24-methylenelanost-8-ene-7,11-dione. These results suggested that there was a hydroxyl group at C-15. The location of the hydroxyl group at C-15 was confirmed by the HMBC correlation of H-15 to C-30. The gross structure of **2** was further confirmed by ^1^H-^1^H COSY and HMBC spectra (Figure 2).

**Figure 4:**
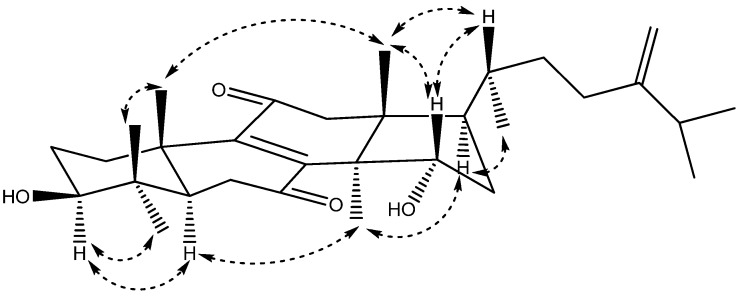
Key NOESY correlations of **2**.

The relative configuration of **2** was determined by extensive analysis of the coupling constant in ^1^H-NMR (Table 1) and the NOESY correlations (Figure 4). The large coupling constant (*J* = 11.2 Hz) between H-3 and H*_β_*-2 clearly indicated that the 3-OH group was in equatorial *β*-position. The NOESY correlations (Figure 2) of H-3 with H-5 and H_3_-28, H_3_-30 with H-5 and H-17, H-17 with H_3_-21 indicated that H-3, H-5, H-17, H_3_-21, H_3_-28, and H_3_-30 were all in *α*-orientation, whereas H_3_-18 with H-15, H_3_-19 and H-20, H_3_-19 with H_3_-29, and H-20 with H-15, exhibited that H-15, H_3_-18, H_3_-19, H-20, and H3-29 were in β-stereochemistry. Thus compound 2 was elucidated as 15α-hydroxyeuphorbol-7,11-dione and the relative configuration of **2** was as shown in Figure 4.

The structures of the known compounds euphorbol-7-one (**3**) [8], friedelin (**4**) [9], stigmast-4-ene-6*α*-ol-3-one (**5**) [10], and stigmasta-4-en-3,6-dione (**6**) [11] were determined by comparison of their spectroscopic data, MS, and optical rotations with those in the literature.

All compounds were examined for antibacterial activity against six terrestrial pathogenic bacteria, including *Staphylococcus aureus*, *Micrococcus luteus*, *Micrococcus tetragenus*, *Staphylococcus albus*, *Bacillus cereus* and *Escherichia coli* by the microplate assay method. The results demonstrated that compounds **4** and **5** showed antibacterial activities against *E. coli* and *M. tetragenus* with the same MIC values of 5 μg/mL. Ciprofloxacin was used as a positive control with the same MIC values of 0.313 μg/mL to against *E. coli* and *M. tetragenus*.

## 3. Experimental

### 3.1. General Information

IR spectra were recorded on a Thermo Nicolet 6700 (using KBr disks) spectrophotometer (Thermo Scientific, Madison, WI, USA). NMR spectra were measured on a Bruker AV-400 instrument with TMS as the internal standard. HRESIMS spectra were made on the Bruker Daltonics Apex-Ultra 7.0 T (Bruker Corporation, Billerica, MA, USA). Silica gel used for column chromatography (CC) was supplied by Qingdao Marine Chemical Factory (Qingdao, China). Sephadex LH-20 was used (Pharmacia Co., Ltd., Sandwich, UK). TLC and preparative TLC were purchased from Qingdao Chemical Industry Institute (Qingdao, China). All solvents were purchased from Tianjin Fuchen Chemical Reagent Factory (Tianjin, China). The optical density was measured by an enzyme-labeled detector (Elx800, BioTek Instruments, lnc., Winooski, VT, USA).

### 3.2. Plant Material

The branches and leaves of *Polyalthia oblique* were collected in Changjiang County, Hainan Province, China, in June 2012 the fruiting season and authenticated by associate Prof. Qiong-Xin Zhong, College of Life Science, Hainan Normal University. They were not separated. The averaged diameter of the collected branches were about 2 cm. A voucher specimen was deposited in the Key Laboratory of Tropical Medicinal Plant Chemistry of Ministry of Education, Hainan Normal University.

### 3.3. Extraction and Isolation

The air-dried and powdered branches and leaves of *Polyalthia oblique* (20 kg) were extracted with 80% EtOH at 50 °C (3 × 6 h). The extracts were then suspended in 2 L water and then partitioned successively with petroleum ether (60–90 °C) and ethyl acetate. These two fractions were designated as petroleum ether portion and ethyl acetate portion, respectively. The petroleum ether portion (180.7 g) was subjected to silica gel column chromatography (48–75 mm) and eluted with petroleum ether/EtOAc (*v*/*v*, gradient 100:0–0:100) to afford Frs. 1–12. Fr. 4 (1.1 g) was chromatographed on silica gel eluted with petroleum ether/EtOAc (*v*/*v*, gradient 3:1–1:1) to give subfractions 4a–f. Fr. 4a was passed through Sephadex LH-20 eluted with CHCl_3_/MeOH (*v*/*v*, 1:1), and further purified by silica gel column chromatography eluting with (CHC1_3_/MeOH, *v*/*v*, 15:1) to yield **1** (7.0 mg), along with **2** (8.0 mg) and **3** (22.0 mg). Fr. 6 (1.2 g) was chromatographed on a silica gel column eluted with petroleum ether/EtOAc (*v*/*v*, gradient 1:0–0:1), recrystallized from MeOH to afford **4** (53 mg). Fr. 8 (0.8 g) was chromatographed on a silica gel column eluted with petroleum ether/CHC1_3_/EtOAc (*v*/*v*, 4:1:1) to afford five major subfractions, Fr.8a–Fr. 8e. Fr. 8b (0.3 g) was further purified on a silica gel column eluted with (CHC1_3_/EtOAc, *v*/*v*, 5:1) to obtain **5** (6.0 mg) and **6** (9.0 mg).

### 3.4. Characterization of Compounds **1**–**2**

*20-Hydroxyeuphorbol-7-one* (**1**): White amorphous powder (7.0 mg); IR (KBr): 3439, 2360, 1642, 1398, 1094, 765, 472 cm^−1^; ^1^H-NMR and ^13^C-NMR: Table 1; ESIMS *m*/*z*: 471.4 [M+H]^+^; HRESIMS *m*/*z* [M+H]^+^: 471.3826 (calcd for C_31_H_51_O_3_, 471.3833).

*15α-Hydroxyeuphorbol-7,11-dione* (**2**): White amorphous powder (8.0 mg); IR (KBr): 3441, 1629, 1398, 1088, 475 cm^−1^; ^1^H-NMR and ^13^C-NMR: Table 1; ESIMS *m*/*z*: 507.6 [M+Na]^+^; HRESIMS *m*/*z* [M+H]^+^: 485.3627 (calcd for C_31_H_49_O_4_, 485.3625).

### 3.5. Antimicrobial Activity Assay

Antimicrobial activity against six bacterial strains *Staphylococcus aureus* (ATCC 8032), *Micrococcus luteus* (ATCC 4698), *Micrococcus tetragenus* (ATCC 13623), *Staphylococcus albus* (ATCC 8032), *Bacillus cereus* (ACCC 11077) and *Escherichia coli* (ATCC 25922), were assayed using standard agar diffusion tests [12]. Ciprofloxacin was used as a positive control.

## 4. Conclusions

In conclusion, our phytochemical investigation of the branches and leaves of *P. oblique* afforded two new triterpenoids, 20-hydroxyeuphorbol-7-one (**1**); and 15-hydroxyeuphorbol-7,11-dione (**2**); together with four known triterpenoids, euphorbol-7-one (**3**); friedelin (**4**); stigmast-4-ene-6*α*-ol-3-one (**5**); stigmasta-4-en-3,6-dione (**6**). Compounds **4** and **5** showed antibacterial activities against *E. coli* and *M. tetragenus* with the same MIC values of 5 μg/mL.

## Supplementary Materials

Supplementary materials can be accessed at: http://www.mdpi.com/1420-3049/19/6/7621/s1.
