# Wang L.K., *et al.* Two New Lanostane Triterpenoids from the Branches and Leaves of *Polyalthia oblique. Molecules* 2014, *19*, 7621–7628

**DOI:** 10.3390/molecules201119690

**Published:** 2015-11-11

**Authors:** Liu-Kai Wang, Cai-Juan Zheng, Xiao-Bao Li, Guang-Ying Chen, Chang-Ri Han, Wen-Hao Chen, Xiao-Ping Song

**Affiliations:** Key Laboratory of Tropical Medicinal Plant Chemistry of Ministry of Education, College of Chemistry and Chemical Engineering, Hainan Normal University, Haikou 571158, Hainan, China

The authors wish to make the following correction to their paper [1], published recently in *Molecules*. The names of compounds **1** and **2** were not correct, and there were some minor errors in the chemical structure of compound **3** shown in Figure 1 of this paper [1]. The names of compounds **1** and **2** should be changed to (3β,20β)-3,20-dihydroxy-24-methylenelanost-8-ene-7-one (**1**) and (3β,15α)-3,15-dihydroxy-24-methylenelanost-8-ene-7,11-dione (**2**). The corrected structure of **3** is shown in Figure 1.

The manuscript will be updated and the original will remain online on the article webpage.

**Figure 1:**
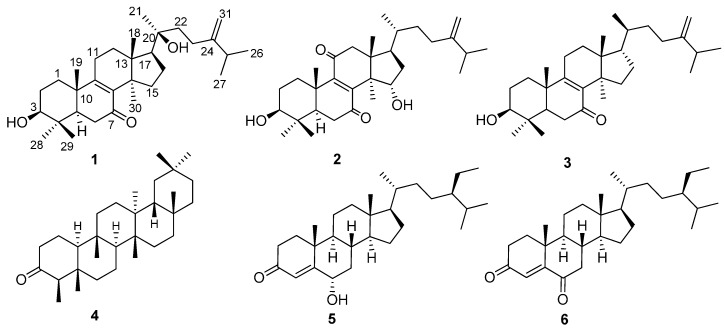
Structures of compounds **1**–**6**.
