# Supplementary material for: Two New Lanostane Triterpenoids from the Branches and Leaves of Polyalthia oblique
Source: Molecules. 2014 Jun 6;19(6):7621–8. doi: 10.3390/molecules19067621 (PMC6271888; doi:10.3390/molecules19067621)
Supplement: Supplementary file 1 [file molecules-19-07621-s001.pdf]

Supporting Information

Figure S1. IR spectrum of compound **1**.

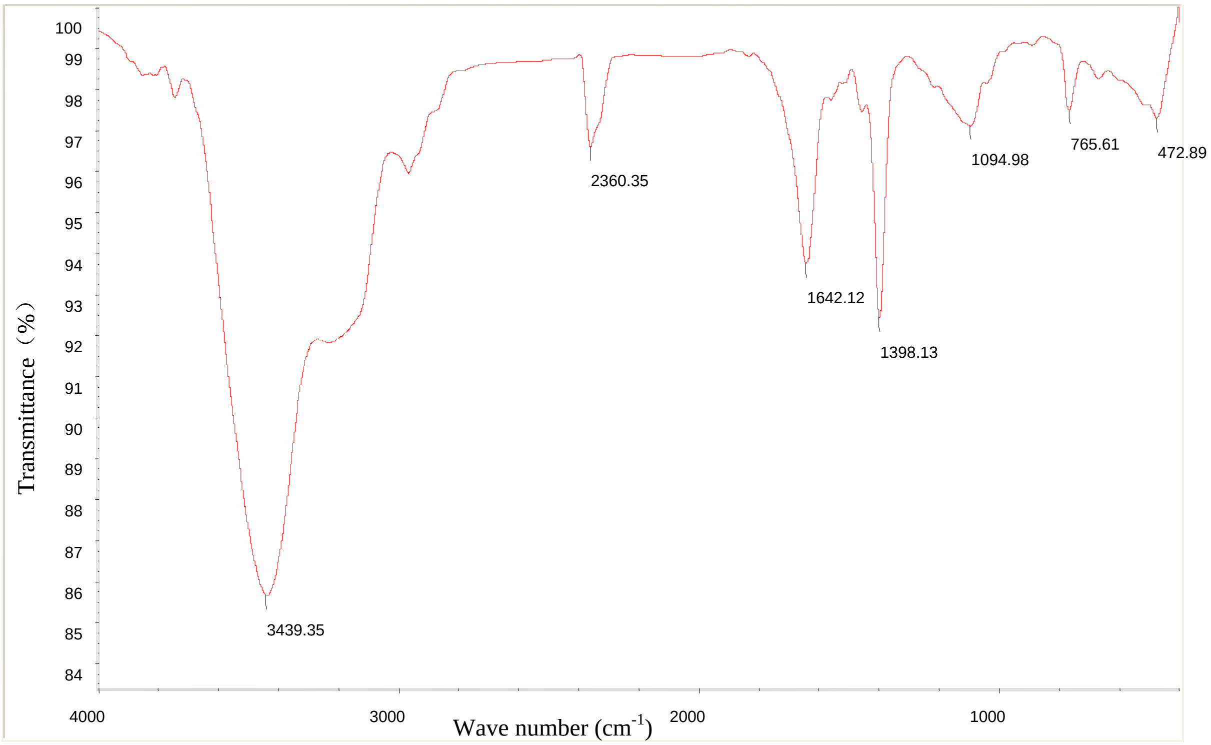

Figure S2. ^{1}H-NMR (400 MHz, $CDCl_3$) spectrum of compound **1**.

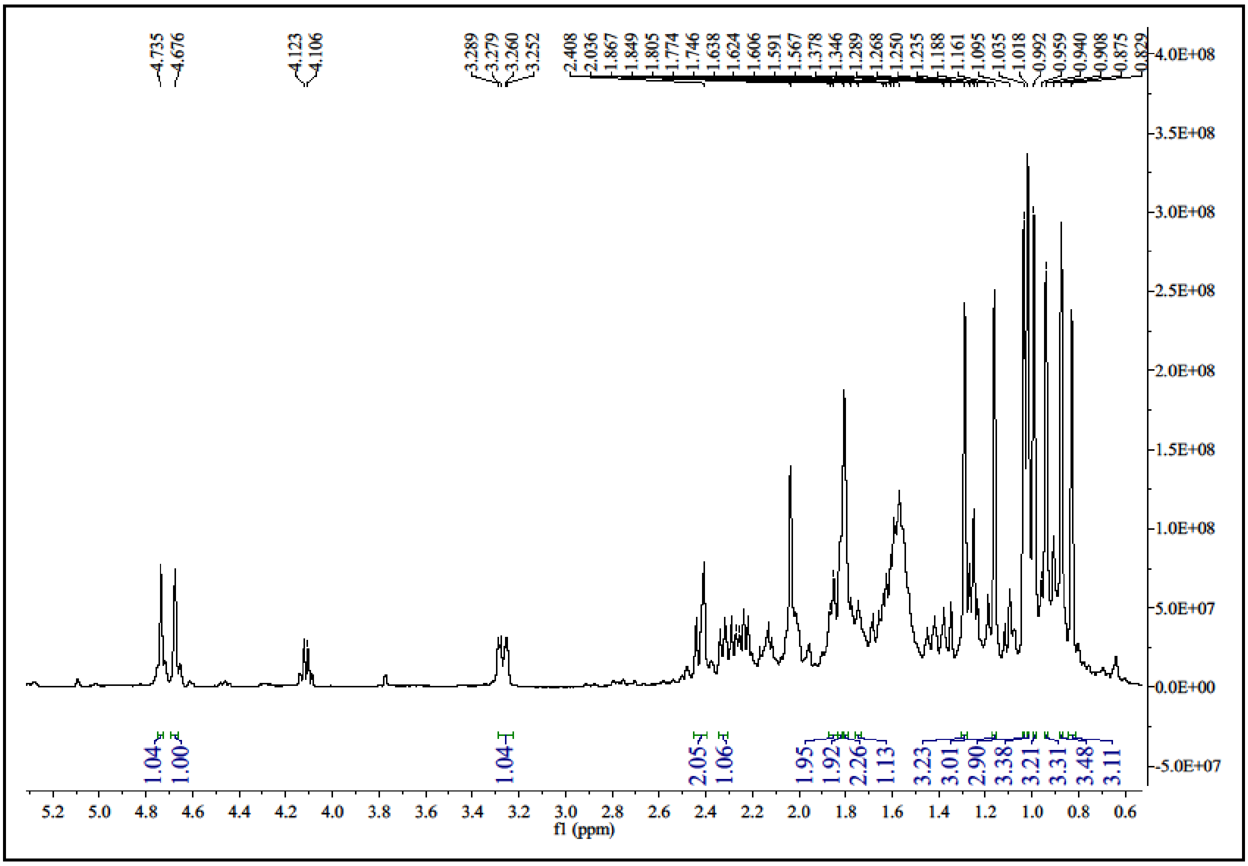

Figure S3. ^{13}C-NMR (100 MHz, $CDCl_3$) spectrum of compound **1**.

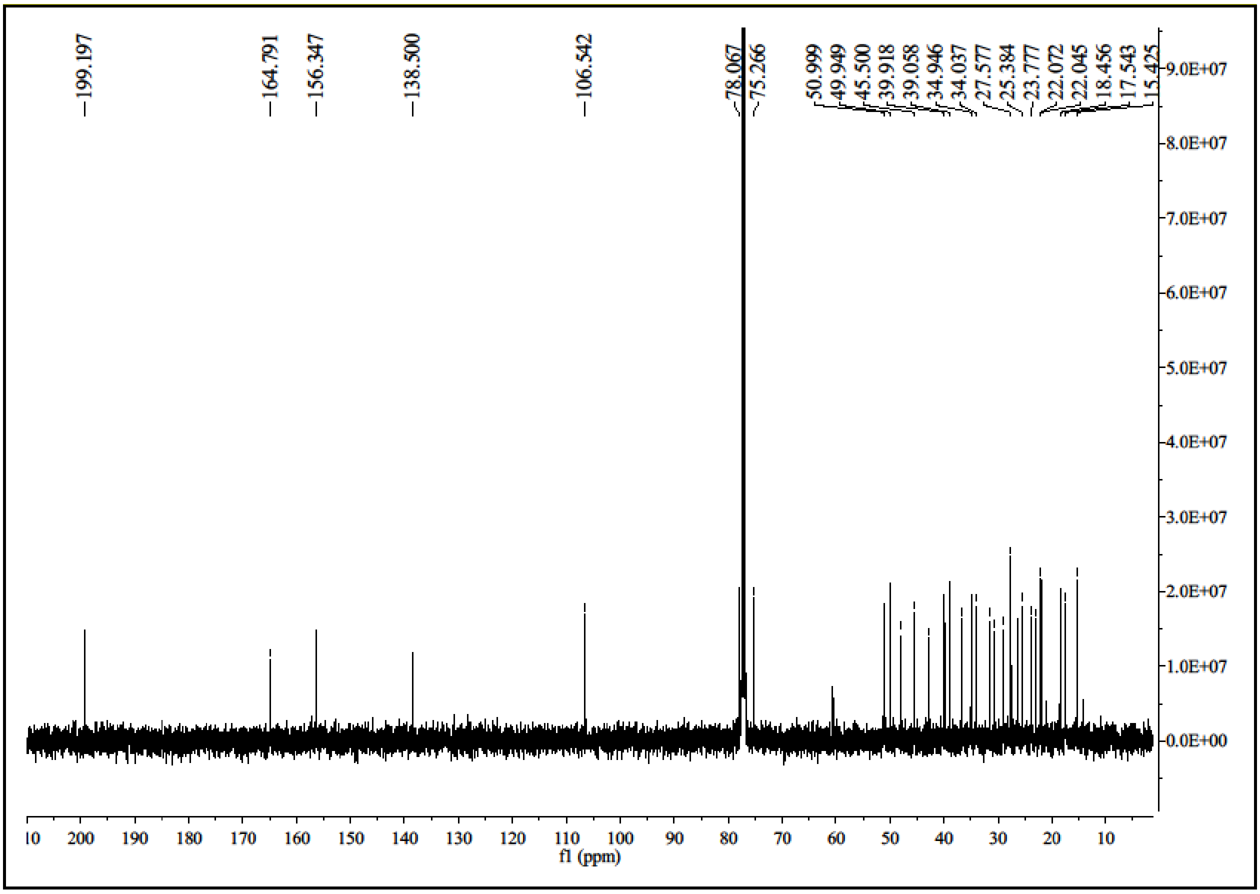

Figure S4. HMQC ($CDCl_3$) spectrum of compound 1.

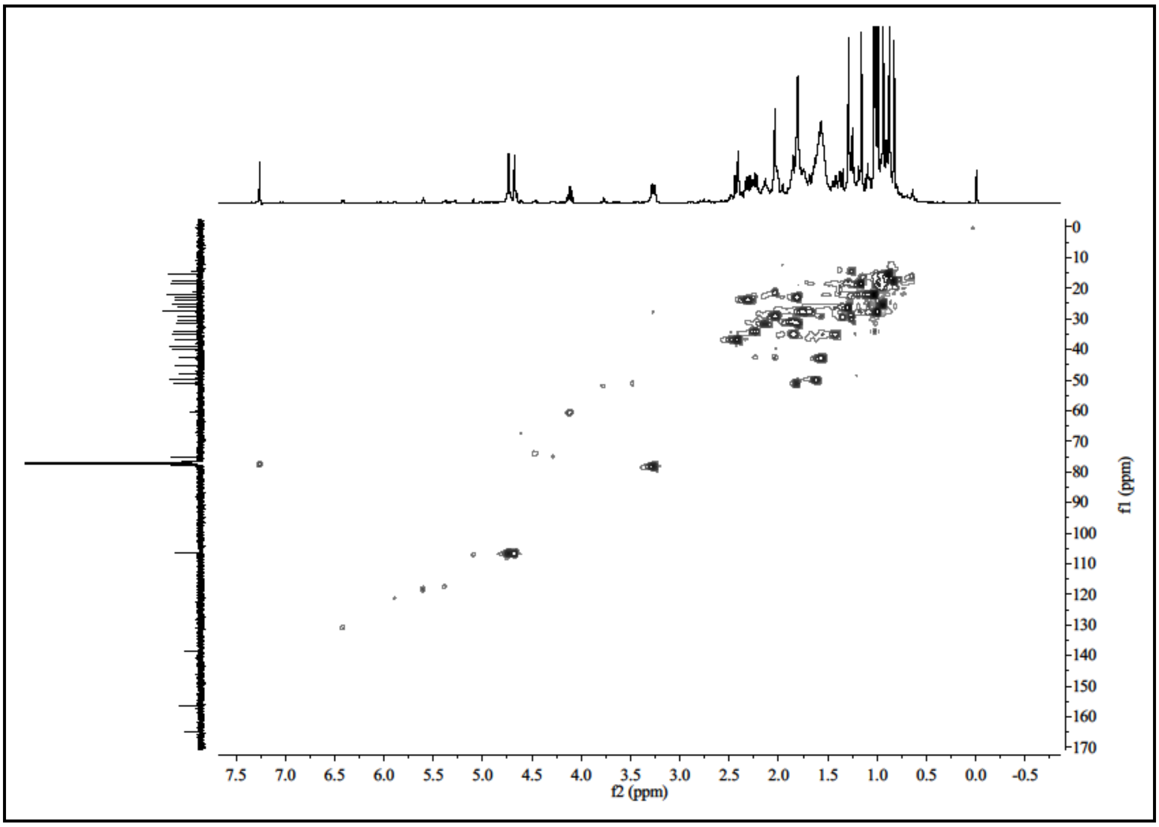

Figure S5. ^1H-^1H COSY ($CDCl_3$) spectrum of compound **1**.

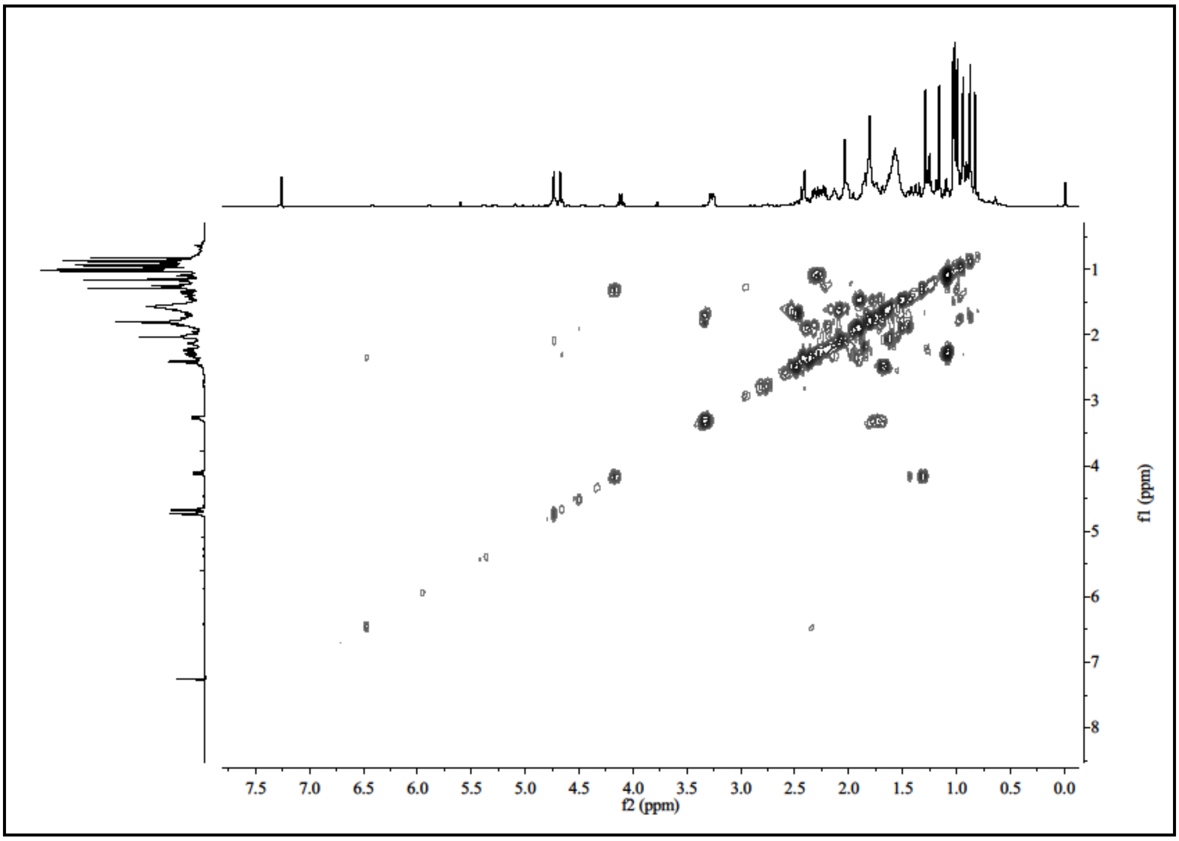

Figure S6. HMBC spectrum ($CDCl_3$) of compound **1**.

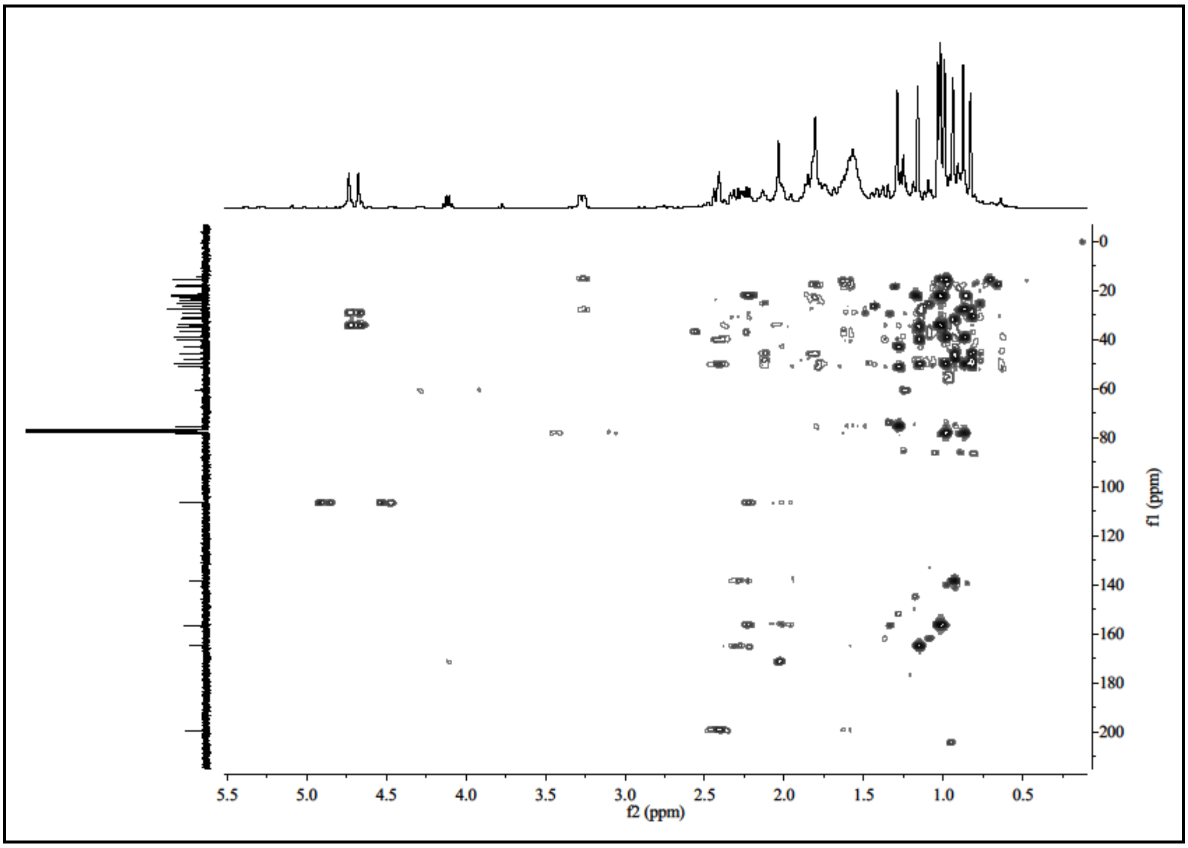

Figure S7. NOESY spectrum of compound **1**.

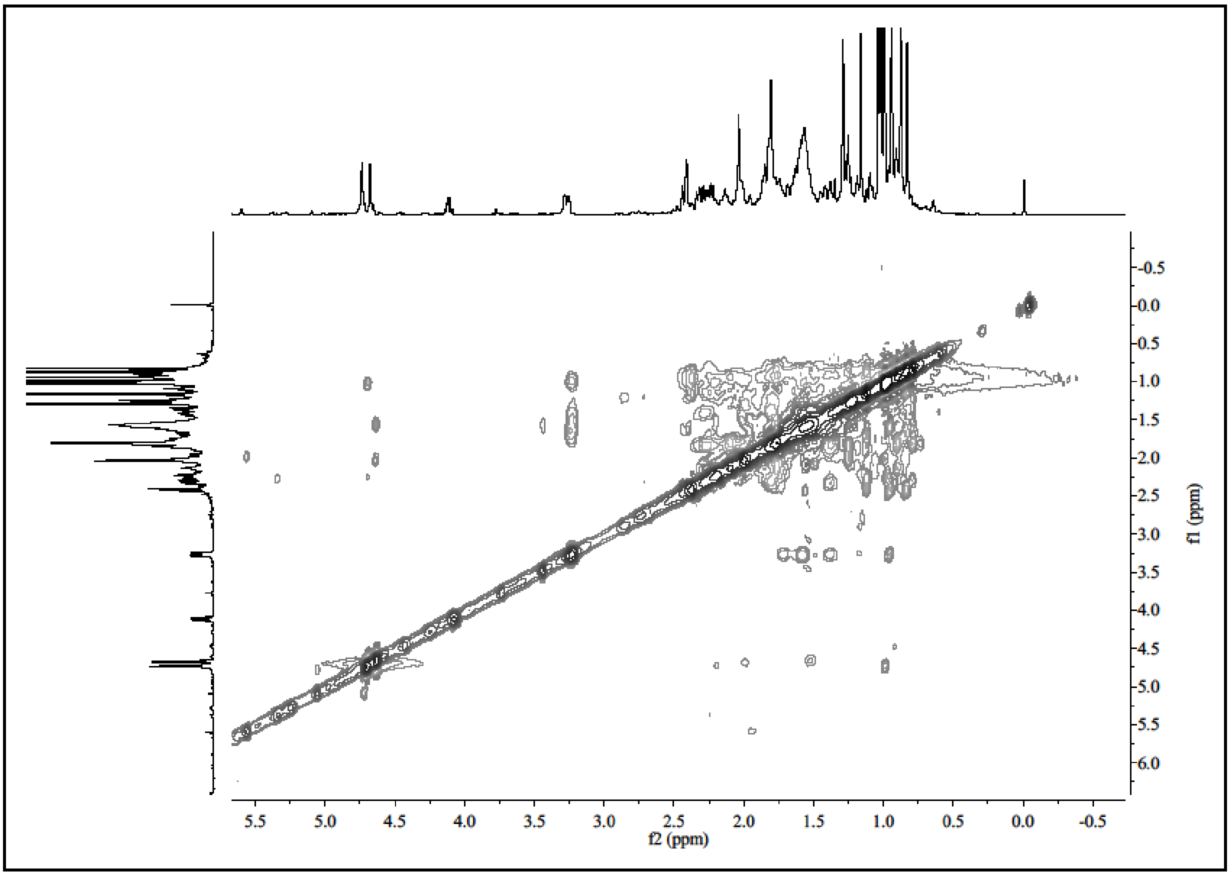

Figure S8. HRESIMS spectrum of compound **1**.

Mass Spectrum SmartFormula Report

Analysis Info

Analysis Name	D:\Data\20130508-1\WLK-33-ESI-POS_000002.d	Acquisition Date	2013/5/8 16:36:09
Method	LC_LANGDU_20130501	Operator	
Sample Name	0	Instrument	apex-Ultra
Comment			

Acquisition Parameter

Polarity	Positive	Source	ESI	No. of Laser Shots	20
Averaged Scans	1	No. of Cell Fills	1	Laser Power	51.0 %
Broadband Low Mass	100.3 m/z	End Plate	3500.0 V	MALDI Plate	300.0 V
Broadband High Mass	800.0 m/z	Capillary Entrance	4000.0 V	Imaging Spot Diameter	2000.0 µm
Acquisition Mode	Single MS	Skimmer 1	20.0 V		
Pulse Program	basic	Drying Gas Temperature	180.0 °C	Calibration Date	Wed May 8 04:13:41
Source Accumulation	0.0 sec	Drying Gas Flow Rate	4.0 L/min	Data Acquisition Size	[illegible]
Ion Accumulation Time	0.1 sec	Nebulizer Gas Flow Rate	1.0 L/min	Apodization	Sine-Bell Multiplication
Flight Time to Acq. Cell	0.0 sec				

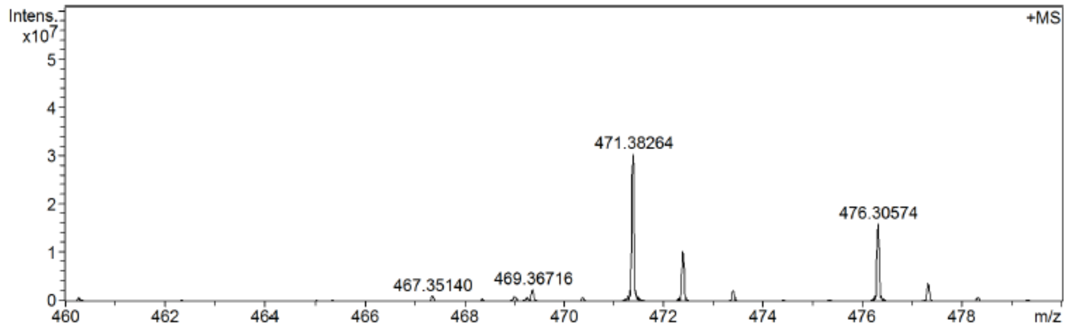

Meas. m/z	#	Formula	Score	m/z	err [mDa]	err [ppm]	mSigma	rdb	e¯ Conf	N-Rule
471.38264	1	C 31 H 51 O 3	100.00	471.38327	0.6	1.3	6.8	6.5	even	ok

Figure S9. IR spectrum of compound **2**.

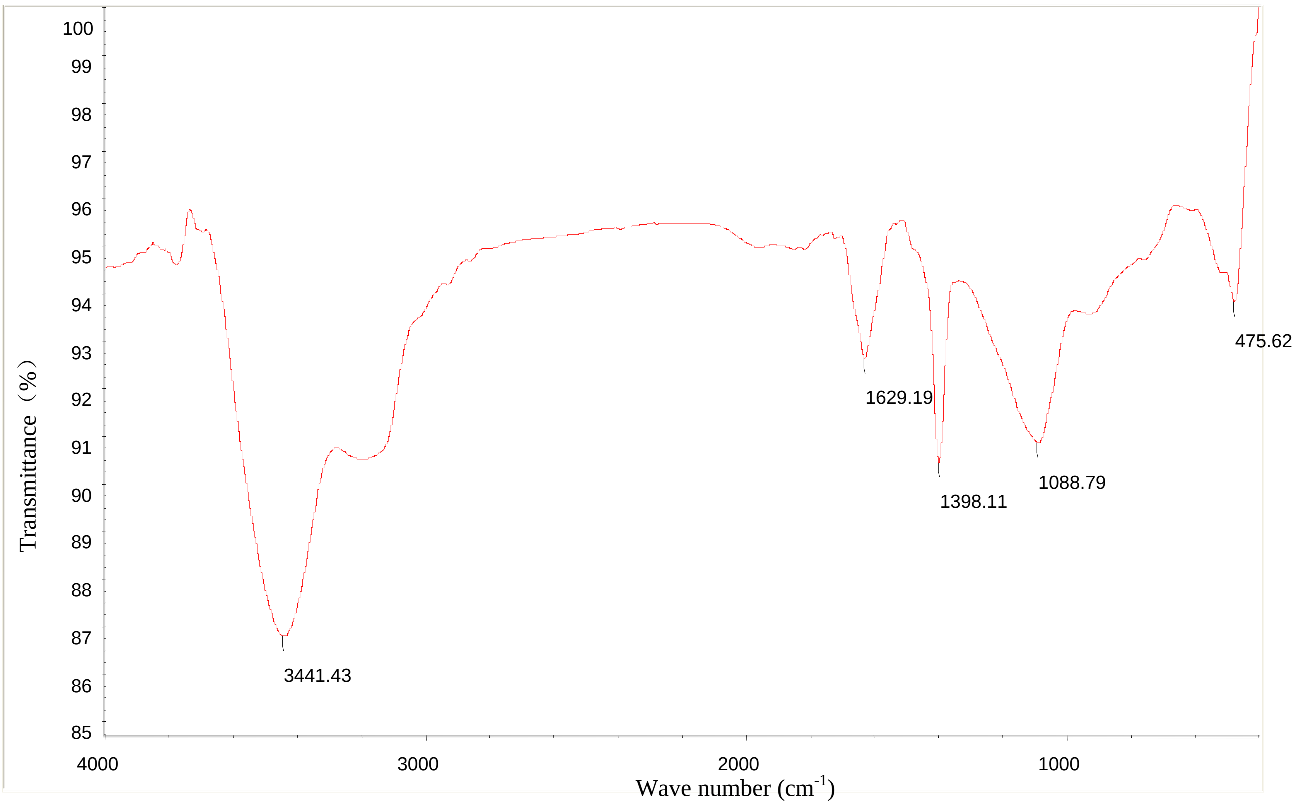

Figure S10. ^{1}H-NMR (400 MHz, $CDCl_3$) spectrum of compound **2**.

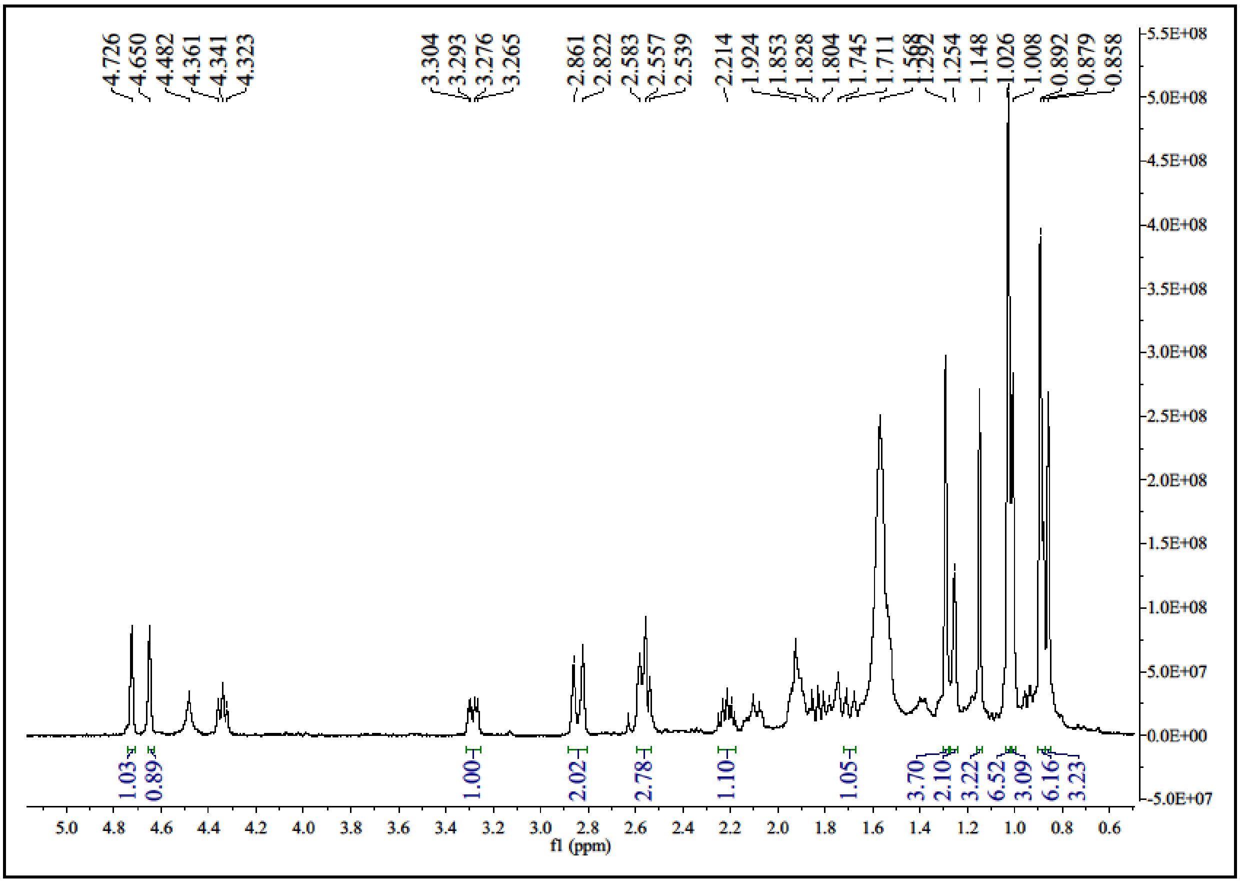

Figure S11. ^{13}C-NMR (100 MHz, $CDCl_3$) spectrum of compound **2**.

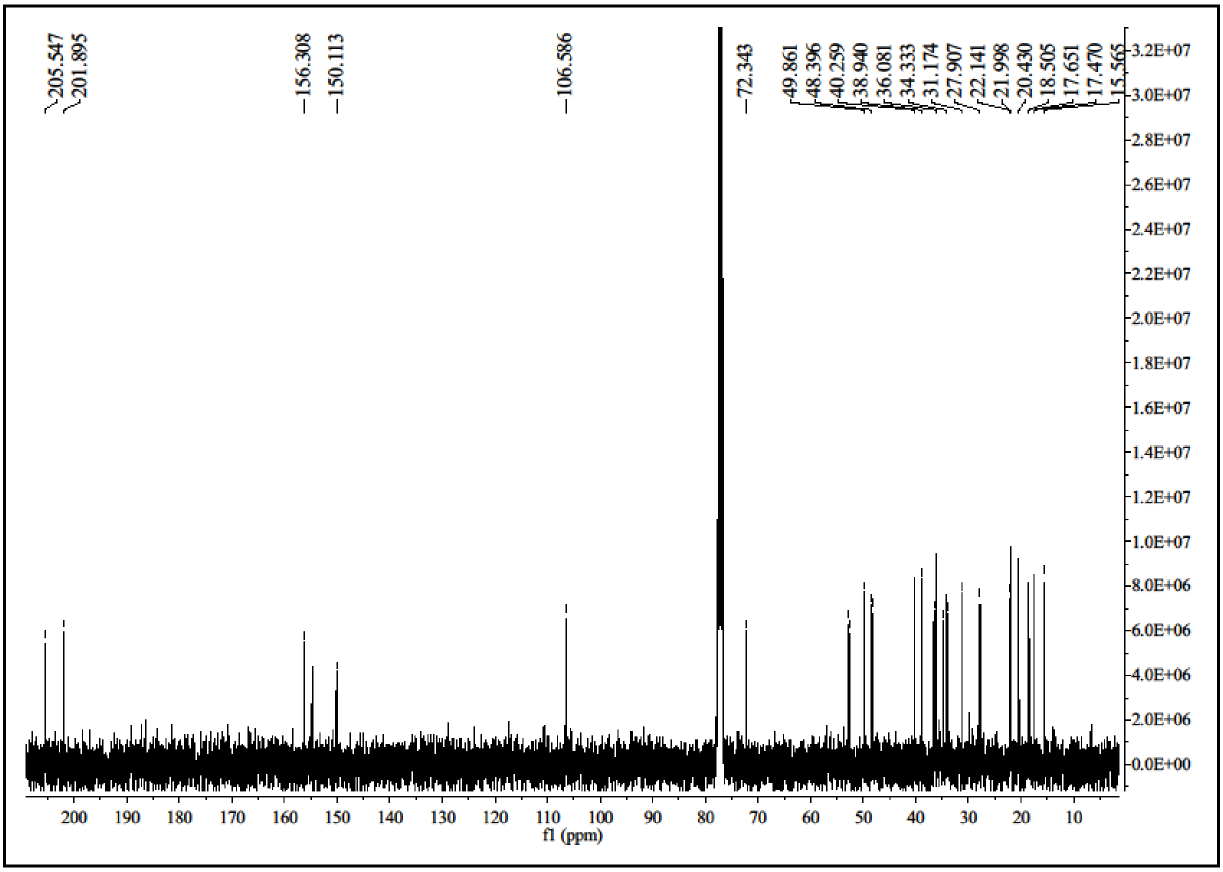

Figure S12. HMQC ($CDCl_3$) spectrum of compound **2**.

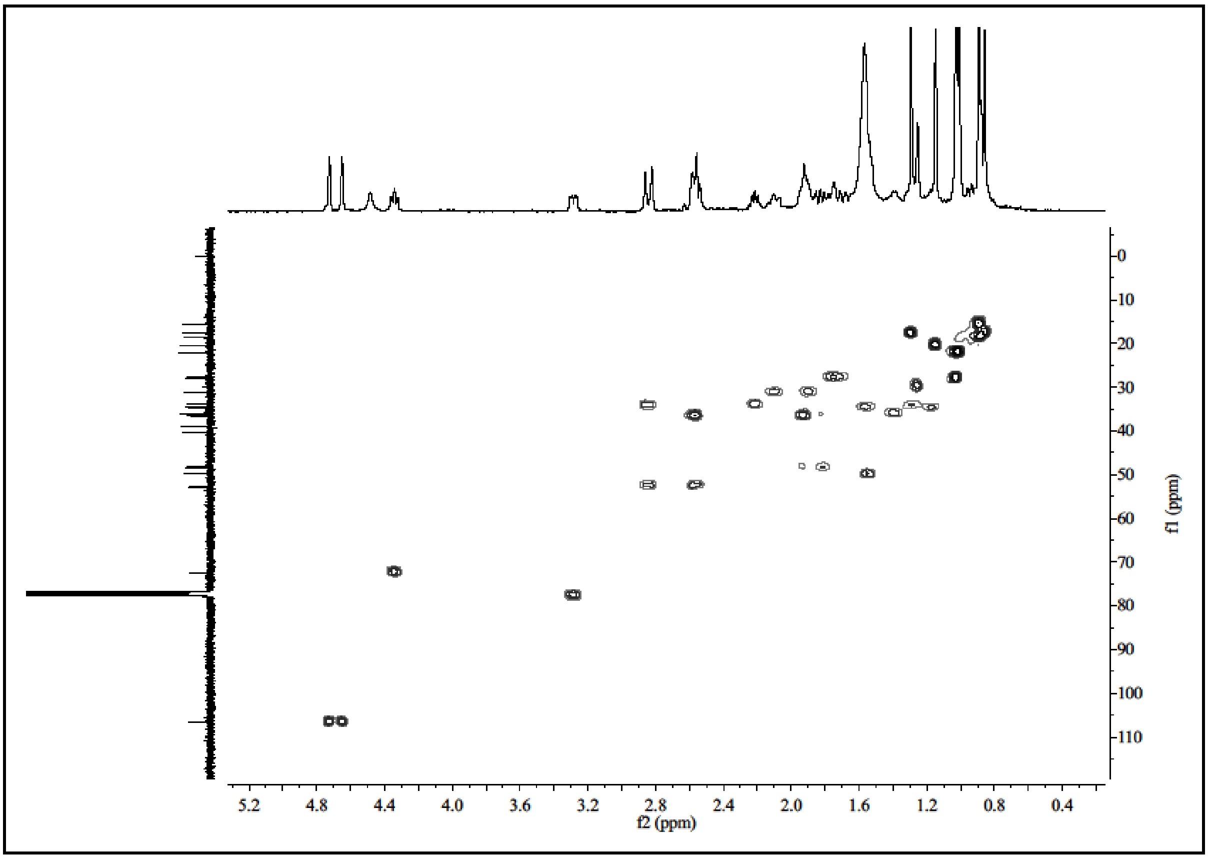

Figure S13. ^{1}H-^{1}H COSY ($CDCl_3$) spectrum of compound **2**.

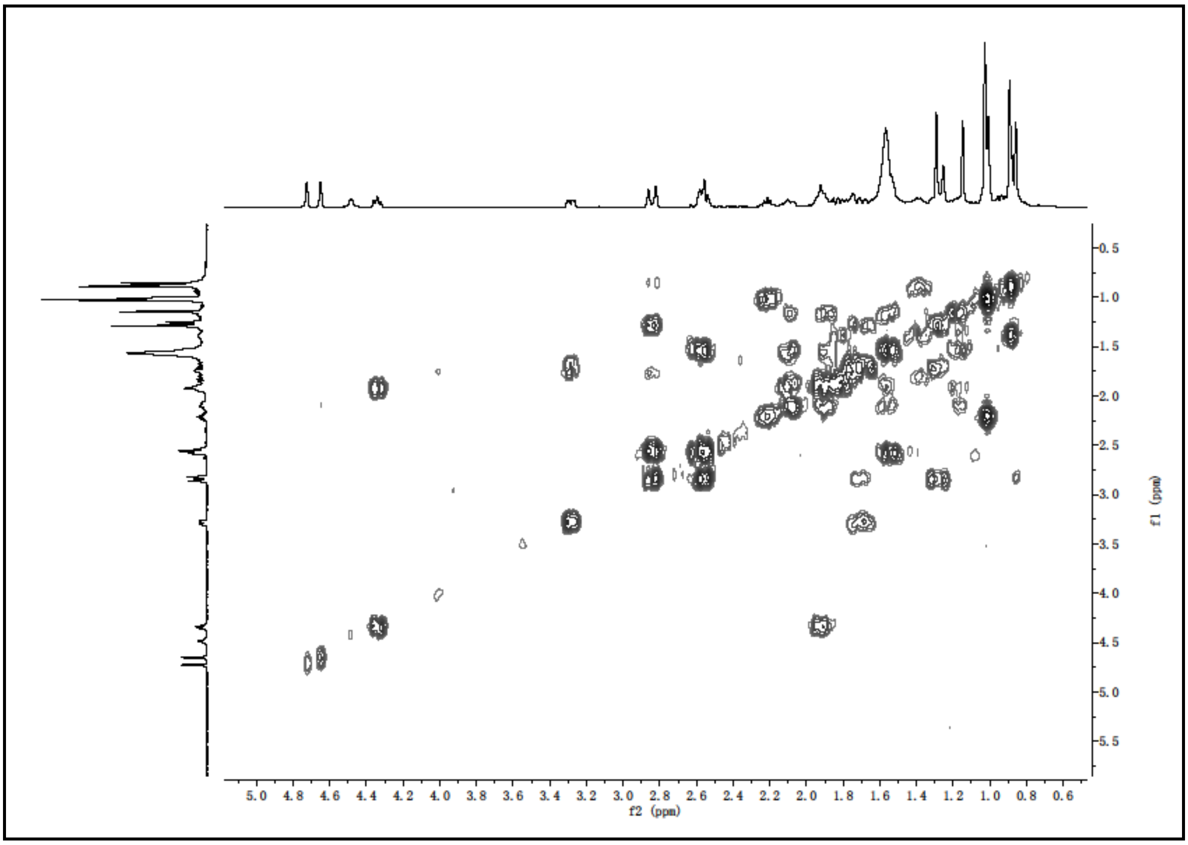

Figure S14. HMBC ($CDCl_3$) spectrum of compound **2**.

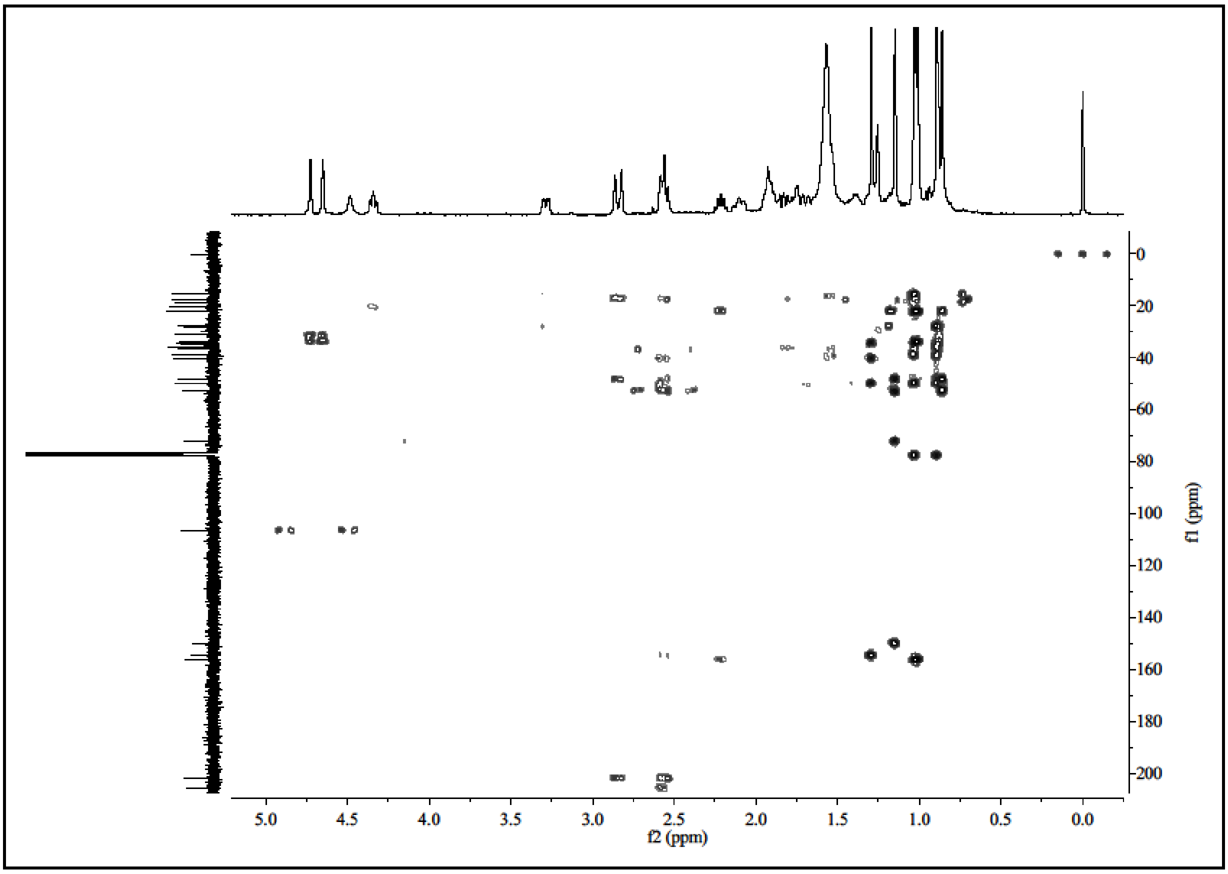

Figure S15. NOESY spectrum of compound **2**.

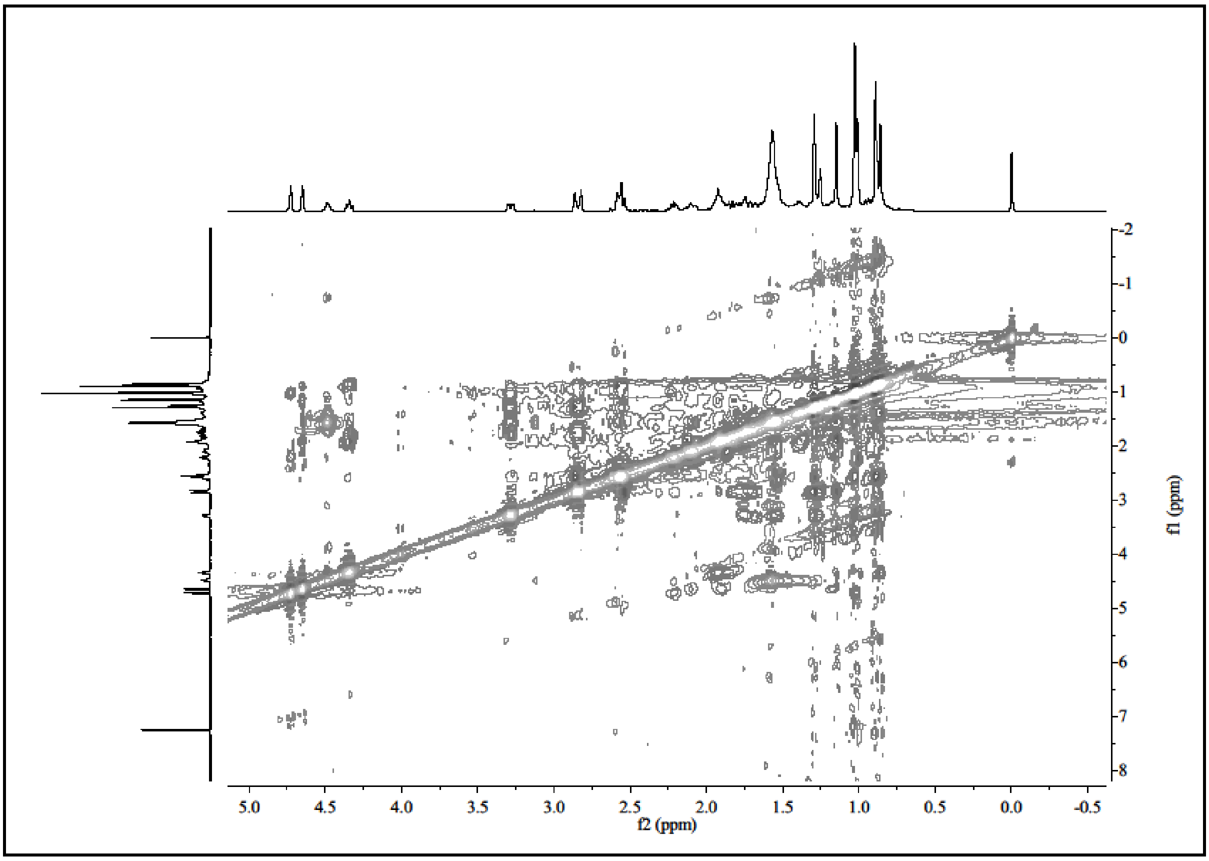

Figure S16. HRESIMS spectrum of compound **2**.

Mass Spectrum SmartFormula Report

Analysis Info

Analysis Name	D:\Data\20130508-1\WLK-20-ESI-POS_000002.d	Acquisition Date	2013/5/8 16:32:59
Method	LC_LANGDU_20130501	Operator	
Sample Name	0	Instrument	apex-Ultra
Comment			

Acquisition Parameter

Polarity	Positive	Source	ESI	No. of Laser Shots	20
Averaged Scans	1	No. of Cell Fills	1	Laser Power	51.0 %
Broadband Low Mass	100.3 m/z	End Plate	3500.0 V	MALDI Plate	300.0 V
Broadband High Mass	800.0 m/z	Capillary Entrance	4000.0 V	Imaging Spot Diameter	2000.0 µm
Acquisition Mode	Single MS	Skimmer 1	20.0 V		
Pulse Program	basic	Drying Gas Temperature	180.0 °C	Calibration Date	Wed May 8 04:13:41
Source Accumulation	0.0 sec	Drying Gas Flow Rate	4.0 L/min	Data Acquisition Size	[illegible]
Ion Accumulation Time	0.1 sec	Nebulizer Gas Flow Rate	1.0 L/min	Apodization	Sine-Bell Multiplication
Flight Time to Acq. Cell	0.0 sec				

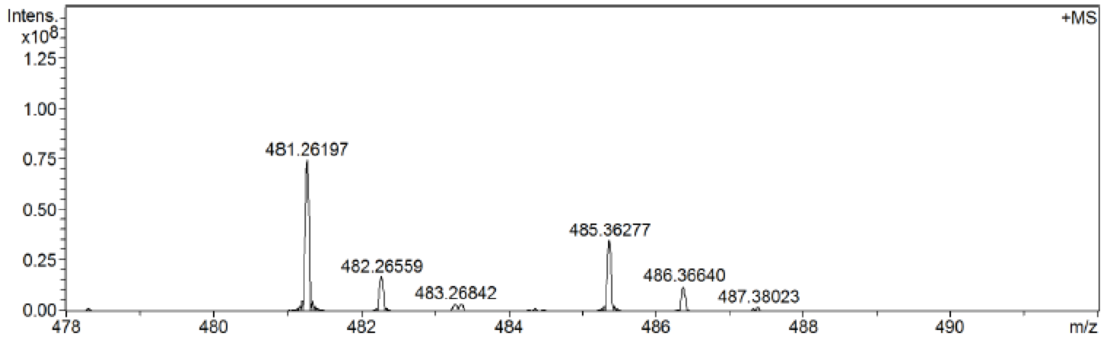

Meas. m/z	#	Formula	Score	m/z	err [mDa]	err [ppm]	mSigma	rdb	e¡¥Conf	N-Rule
485.36277	1	C 31 H 49 O 4	100.00	485.36254	-0.2	-0.5	9.9	7.5	even	ok
